# The influence of chalk grasslands on butterfly phenology and ecology

**DOI:** 10.1002/ece3.8111

**Published:** 2021-10-07

**Authors:** Matthew P. Greenwell, Marc S. Botham, Michael W. Bruford, John C. Day, Luke C. Evans, Melanie Gibbs, Ian Middlebrook, David B. Roy, Kevin Watts, Tom H. Oliver

**Affiliations:** ^1^ School of Biological Sciences University of Reading Reading UK; ^2^ UK Centre for Ecology and Hydrology Wallingford UK; ^3^ School of Biological Sciences Cardiff University Cardiff UK; ^4^ Butterfly Conservation Dorset UK; ^5^ Forest Research Farnham UK

**Keywords:** chalk grassland, drought tolerance, *Maniola jurtina*, meadow brown, phenology, population genetics

## Abstract

The influence of large‐scale variables such as climate change on phenology has received a great deal of research attention. However, local environmental factors also play a key role in determining the timing of species life cycles. Using the meadow brown butterfly *Maniola jurtina* as an example, we investigate how a specific habitat type, lowland calcareous grassland, can affect the timing of flight dates. Although protracted flight periods have previously been reported in populations on chalk grassland sites in the south of England, no attempt has yet been made to quantify this at a national level, or to assess links with population genetics and drought tolerance. Using data from 539 sites across the UK, these differences in phenology are quantified, and *M*. *jurtina* phenology is found to be strongly associated with both site geology and topography, independent of levels of abundance. Further investigation into aspects of *M*. *jurtina* ecology at a subset of sites finds no genetic structuring or drought tolerance associated with these same site conditions.

## Graphical abstract

*Maniola jurtina* phenology is found to be strongly associated with both site geology and topography, independent of mean abundance across 539 monitoring sites. Across a subset of sites, no genetic structuring was associated with these site characteristics. Populations displayed high levels of genetic diversity but low genetic differentiation. Across a subset of sites, drought tolerance was not associated with site geology.

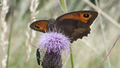

## 1 INTRODUCTION

Changes in the phenology of Lepidoptera is a well‐studied subject (Dell et al., 2005; Diamond et al., 2011; Hodgson et al., 2011; MacGregor et al., 2019; Roy et al., 2015; Roy & Sparks, 2000) in part because long‐term monitoring data are available for a large number of species, allowing temporal changes in phenology to be measured (Roy & Sparks, 2000). Phenology, the annual timing of species life cycles, can be affected by a host of environmental factors such as temperature and daylength (Bale et al., 2002). For example, increasing temperatures as a result of climate change have been shown to shift Lepidoptera phenology by advancing first flight dates (Roy & Sparks, 2000). Temperature varies spatially and temporally, resulting in changes in phenology based upon latitude. For example, butterfly flight periods have been shown to be shorter and begin later at northern latitudes (Brakefield, 1987). However, equivalent changes to environmental factors occurring spatially and temporally can have different effects on phenology, with temporal changes in annual temperatures in fixed locations affecting phenology to a greater degree than differences in temperature spatially (Doi & Takahashi, 2008; Roy et al., 2015). This suggests that species may have some level of local adaptation as well as responding to fixed environmental conditions (Hodgson et al., 2011; Roy et al., 2015).

**FIGURE 1:**
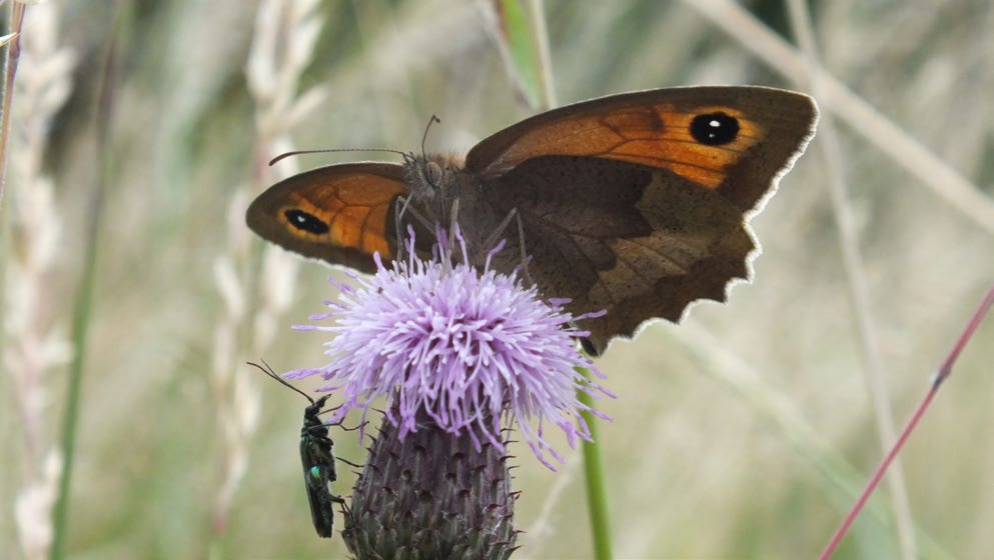
Meadow brown butterfly, *Maniola jurtina*

Less attention has been paid to the effects of local, site‐specific, environmental variation on phenology. In this study, we focus on how site‐specific characteristics affect the phenology of the Lepidoptera *Maniola jurtina* (Figure 1), a species known to be particularly affected by local site conditions, with protracted flight periods and occasional second peaks in emergence observed on chalk grasslands in the south of England (Goulson, 1993a; Thomas & Lewington, 2010). *M*. *jurtina* is one of the most common and widely distributed butterfly species in Europe. Found in open grassland habitats (Schmitt et al., 2005), on average, adult *M*. *jurtina* individuals move around an area with a radius of 320 m; however, in mark–release–recapture studies, individuals have been found up to 2.1 km away from where they were released (Schneider et al., 2003). The phenology of *M*. *jurtina* is unusually long for a univoltine, grassland species in the UK, with adults typically on the wing from mid‐June to September (Thomas & Lewington, 2010).

Although the protracted flight period of *M*. *jurtina* on chalk grasslands in the UK has received previous investigation (Goulson, 1993a; Shreeve, 1989), no effort has yet been made to quantify these differences in phenology at the national scale. Compared with landscapes such as farmland or woodland, chalk grasslands are warmer and drier during summer, resulting in more favorable conditions for thermophilic species (Mortimer et al., 1998). They are also more topographically heterogeneous (Diacon‐Bolli et al., 2012; Mortimer et al., 1998) due to differences in vegetation structure and topography, resulting in variation in ground temperatures (Maclean et al., 2019). The resulting microclimates may allow individuals to persist in specific locations when surrounding areas of habitat are climatically unsuitable (Bennie et al., 2008; Suggitt et al., 2011), potentially broadening the flight period. Similarly, extreme warm temperatures in some microclimates may result in local drought conditions which are likely to affect larval development, for example, larvae of speckled wood (*Pararge aegeria*) reared on drought‐stressed plants show longer development times and increased mortality rates (Gibbs et al., 2012, 2018; Talloen et al., 2004). Thus, longer development times for some individuals and climatically suitable patches may both contribute to the longer flight periods. Protracted flight periods are also observed in some *M*. *jurtina* populations in southern Europe (Haeler et al., 2014); however, this results from adult females entering a period of aestivation (Brakefield, 1984), which has hitherto not been observed in observations of UK populations. Additionally, as this appears to be controlled by geographic provenance and associated larval developmental conditions (Grill et al., 2013), it is unlikely this behavior is present in any UK population.

Although it seems likely that the variation in UK *M*. *jurtina* phenology results from differences in conditions that occur within chalk grasslands, the mechanisms that cause these responses are unclear. A parsimonious explanation of the protracted flight period is that these sites contain more favorable habitat and therefore higher abundances, with the broad flight periods simply a result of the mathematical relationship between mean and variance (Taylor, 1961). If, however, the broad flight period of *M*. *jurtina* on chalk grasslands is not purely the result of high abundances, differences in the local site conditions and the ecology of populations at these sites may be affecting phenology.

The broader flight periods on chalk grasslands may be the result of genetic differences between populations, with some anecdotal suggestions of locally adapted races. Although we do not explicitly look at local adaptation here, we do investigate the potential for genetic structuring between populations, based upon the type of site that individuals are found in. Clear genetic clustering of individuals into chalk and nonchalk populations would suggest a high level of genetic differentiation, which may support the idea of locally adapted races as an explanation for the differences in flight periods.

To explore these possibilities, we examine the flight periods of *M*. *jurtina* in the UK at 539 sites differing in geology and topography and quantify the variability in phenology. We confirm that flight periods are protracted on chalk grasslands across a wide spatial scale, as previously reported at local sites (Goulson, 1993a). After controlling for abundance in our models, we then investigate levels of genetic diversity and differentiation, and drought tolerance at a subset of sites to determine whether differences in phenology are associated with genetic structuring of populations and whether there is evidence of increased drought tolerance from chalk sites that may influence the flight period length. Overall, we test the following:

1. To what extent are *M*. *jurtina* population flight periods protracted on chalk grasslands in the UK?
2. Are populations of *M*. *jurtina* clustered into genetically structured populations based upon the same habitat conditions?
3. Are populations of *M*. *jurtina* on chalk grasslands more drought‐tolerant than populations in other habitats?

## 2 MATERIALS AND METHODS

### 2.1 Long‐term butterfly monitoring sites and landscape context

**FIGURE 2:**
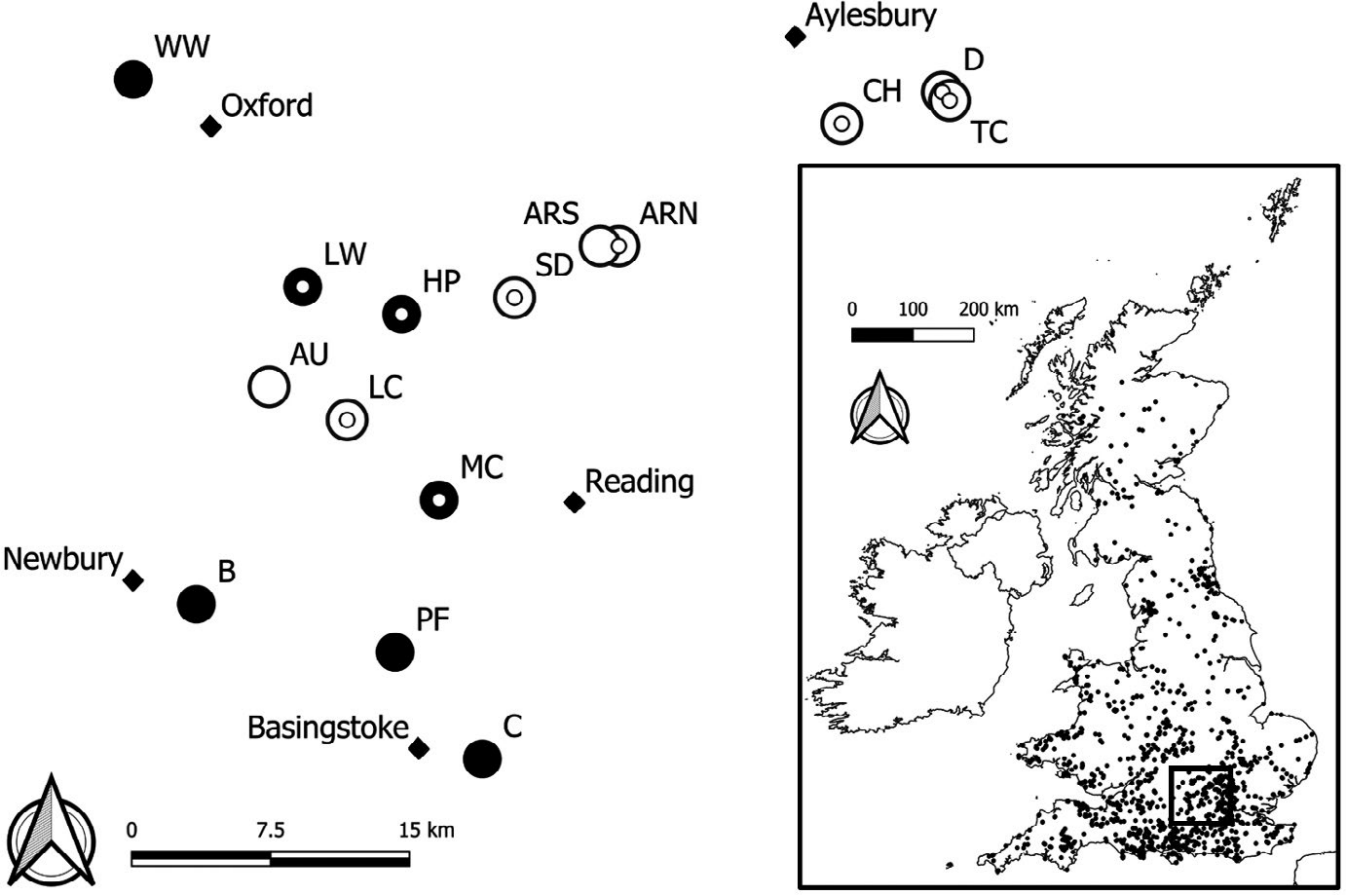
Fifteen sites around the Chiltern Hills from which *Maniola jurtina* samples were collected for genetic analysis. Large black circles signify where no chalk grassland was present within the 500 m radius of each site (*n* = 7), large white circles signify sites where chalk grassland occurred within a 500 m radius of the site centroid (*n* = 8; percentage cover 4.7%–21%). Circles with smaller dots at the centre were sites from which individuals were also collected for the drought experiment (*n* = 9). Main towns are marked with black diamonds. Labelled site names are as follows: Aston Rowant North (ARN), Aston Rowant South (ARS), Aston Upthorpe (AU), Bowdown Forest (B), Crabtree Plantation (C), Coombe Hill (CH), Dancersend (D), Howbery Park (HP), Lardon Chase (LC), Little Wittenham (LW), Moore Copse (MC), Pamber Forest (PF), Swyncombe Down (SD), The Crong (TC), Wytham Woods (WW). Inset map shows the locations of 539 UKBMS transect sites used in the phenology analysis

Abundance data from 539 long‐term monitoring sites (1976 onward; Figure 2) of the UK Butterfly Monitoring Scheme (UKBMS) were used to investigate *M*. *jurtina* phenology. The UKBMS sites were selected if they had both relevant Natural England priority habitat map and digital elevation data (see below). UKBMS data are collected by volunteers using the “Pollard walk” method (Pollard & Yates, 1993). The UKBMS uses a two‐step method (Dennis et al., 2013), using these data to fit generalized additive models which produce fitted weekly counts and an overall collated annual index of abundance at each site (Botham et al., 2020).

To quantify local site characteristics and capture the focal habitat within survey areas, we analyzed a 500‐m radius buffer around the centroid of each of the 539 UKBMS sites, using data from the Natural England priority habitat maps (Natural England, 2019). These maps capture a range of habitat characteristics, including lowland calcareous grassland (chalk grassland). Using a 50‐m resolution digital elevation map (Morris & Flavin, 1990), topographic slope angles were estimated for the 539 UKBMS sites, using a systematic sampling of points at 50‐m intervals within the 500‐m radii of the site centroids, as described in Oliver et al. (2010). It should be noted that site steepness is positively correlated with increased variation in slope angles, that is, areas with steeper slopes are also more topographically variable (see Appendix 1, Tables A1, A2).

For the population genetics analyses, distinct categories of sites were required. Sites were defined as either chalk or nonchalk sites based upon the presence of lowland calcareous grassland (Appendix 1, Table A3). The lowest percentage cover was 4.7% (“Dancersend” site). Although this represents a small percentage of the total site, it is worth noting that few sites across all UKBMS sites where lowland calcareous grassland is present (and associated with extended *M*. *jurtina* phenology from our monitoring data analysis) are dominated (>50% cover) by lowland calcareous grassland and that 25% of these sites (*n* = 70) have less than 4.3% cover. All of the chalk sites used in the analysis fall within the interquartile range of chalk cover across all UKBMS sites.

### 2.2 Drought tolerance experiment

All drought experimentation was carried out following the methodology described in Gibbs et al. (2012). A summary of the methods is provided here. Potted host plants (*Poa trivialis*) were grown under standard conditions, with each plant watered via individual trays. Once mature, plants were randomly assigned to the treatment groups—drought‐stressed or control. Control plants were watered daily from 20 days prior to larval hatching and then throughout the experiment. Plants were never oversaturated but watered enough to prevent soil drying and wilting. Drought‐stressed plants received no water from 20 days prior to larval hatching and were then only watered every six days throughout the experiment. This treatment meant that green leaves were available at all stages of the experiment but ensured moderate drought stress occurred. At the end of the experiment, green leaves were still present on all plants. This ensured that food availability was not a factor limiting larval growth and survival. Rainwater was used in both treatments.

A total of 324 newly hatched *M*. *jurtina* larvae were selected from populations originating from nine of the 15 sites used in the molecular analysis (Figure 2). Adults from these source populations were live‐captured between the 21st of July and 4th of August, mated with individuals from the same population, and eggs were collected. In a common garden experiment, 12 newly hatched larvae from each source population were raised on three non‐drought‐stressed (control) host plants (four larvae, originating from the same source population, per plant) and 24 larvae were raised on six drought‐stressed host plants (four larvae, originating from the same source population, per plant) under controlled conditions until eclosion, using the methods described in Gibbs et al. (2012). A higher number of larvae were raised on drought‐stressed plants due to an expected higher mortality rate (see Talloen et al., 2004), totaling 108 and 216 larvae on control and drought‐stressed host plants, respectively. *M*. *jurtina* overwinter as small larvae, during which little growth occurs (Brakefield, 1984). As such, larvae were monitored at three time points: 49 days after the first larval hatch date (pre‐overwintering), 162 days after hatching (post overwintering during larval growth), and 309 days after hatching (late larval growth and pupation phase). The number of larvae that survived until the third monitoring point was recorded. Individuals were monitored until they reached the pre‐pupa stage, at which point they were removed.

### 2.3 Molecular analysis

We conducted a molecular analysis of 287 *M*. *jurtina* individuals sampled from 15 of the 539 UKBMS sites, comprising eight chalk and seven nonchalk sites around the Chiltern Hills in the south of England between the 10th and 12th of July 2017 (Figure 2). To assess how landscape factors affect gene flow, distances between sites ranged from 0.8 km to 62 km, and intervening landscape encompassed urban areas, arable farmland, woodland, and seminatural habitats. DNA was extracted from a leg of each individual using prepGEM Universal DNA extraction kits (Zygem), following the recommended protocol for insects. Six microsatellite markers, isolated in Richard et al. (2015), were used to genotype the samples: Mj4870, Mj7232, Mj7132, Mj5522, Mj5331, and Mj0247. DNA was amplified in two multiplex sets using the following reaction mixture: 1 μl template DNA, 6.25 μl QIAGEN Multiplex PCR Master Mix (3 mM MgCl_2_), 0.625 μl tagged forward primer, 0.625 μl reverse primer, 1.25 μl QIAGEN Q solution, and 2.25 μl RNase‐free water. Multiplex set 1 contained Mj7232, Mj5522, and Mj0247, all at 3 μM. Multiplex set 2 contained Mj4870 at 1.5 μM, Mj7132 at 5 μM, and Mj5331 at 4.5 μM. PCRs were carried out in an Eppendorf Mastercycler nexus eco with an initial denaturation for 15:00 at 95℃, followed by 40 cycles of 00:30 at 94℃, 01:30 at 56℃, and 01:00 at 72℃, and a final extension 10:00 at 72℃. All PCR products were diluted by 100× and run on an Applied Biosystems 3730 DNA Analyser. Allele peaks were then scored by using GeneMarker®, version 1.5 by SoftGenetics, using the microsatellite calibration settings.

### 2.4 Statistical analysis

#### 2.4.1 Phenology

To calculate butterfly flight periods, all weekly fitted count values for *M*. *jurtina* abundance were summed per UKBMS site in each year, and the day number of the recording period at which 10% of the total occurred was recorded as the flight period start date. The day at which 90% of the total occurred was recorded as the flight period end date. We used 10th and 90th percentiles to avoid the effect of outliers as in WallisDeVries et al. (2011). The flight period was calculated as the number of days between these two values. The mean flight dates for each site per year were also recorded. We note here that the full protraction of the flight period at some sites may not be captured if the flight period continues past the final UKBMS recording date.

We fitted statistical models to understand whether the inferred geology (herein geology) and topography of the site predicted *M*. *jurtina* phenology (Equation 1). The four measures of timing for *M*. *jurtina* flight periods (start, mean, and end dates of the flight period and length of flight period) were each fitted as response variables into separate linear mixed effects models, against the percentage cover of chalk grassland and mean slope angle of each site. The additional factors of mean abundance, northing (km north on Ordnance Survey grid), easting (km east on Ordinance Survey grid), mean site altitude, and mean site aspect (cos((aspect × **π**)/180), such that 1 = due north, −1 = due south) were included as fixed effects and site and year as random effects. We included mean annual abundance as a covariate in these models because larger populations are likely to have a greater flight period range due to mathematical mean–variance relationship (Taylor, 1961). Northing was included in the model to account for the temperature gradient across the UK, with cooler average temperatures occurring at more northerly locations. This was necessary first because previous studies have shown that *M*. *jurtina* flight periods are shorter and begin later at northern latitudes (Brakefield, 1987) and second because temperature has been shown to affect *M*. *jurtina* phenology, with a predicted 4.7 and 5.4 days in advance to the first appearance and peak flight dates, respectively, per 1℃ increase (Roy & Sparks, 2000). Easting was included to account for longitudinal differences in site conditions, for example, differing levels of rainfall which can affect butterfly phenology (Roy et al., 2001). Site altitude and aspect were included to account for the effects these two factors might have on local temperatures. To reduce the range of magnitudes across the data, northing and easting were scaled by subtracting the mean from each value, followed by dividing by the standard deviation. Site and year were included as random effects to account for repeated measures at each site and variation in phenology between years, often associated with weather (Roy & Sparks, 2000).

All mixed‐effects models were carried out in R (R Core Team, 2020) using the *lmer* function from the *lme4* package (Bates et al., 2015). Model assumptions were checked using diagnostic plots for all mixed‐effects models. Diagnostics from the initial model fits demonstrated that phenology at sites with very low abundances was much more variable, violating homoscedasticity. This is likely because at sites with very low abundances, there is increased detectability‐related sampling error, increasing the uncertainty of the phenology estimate (McCarthy et al., 2013). To overcome this problem, all sites with an abundance index value of less than 20 were removed from the analysis.

(1) P=C+S+A+N+E+H+F+i+y+ε,

where *P* is the phenology metric of interest (flight period start, mean, end day, or range), *C* is the percentage cover of chalk grassland per site, *S* is the mean slope angle per site, *A* is the site total abundance, *N* is the site northing, *E* is the site easting, *H* is the mean altitude per site, *F* is the mean aspect of each site, *i* is a random intercept for site, *y* is a random intercept for year, and *ε* indicates error term with zero mean and normal distribution.

All models were tested for spatial autocorrelation via Moran's *I* test. Residuals were extracted from each model and run against an inverse matrix of distance between sampling points using the *Moran.I* function from the *ape* package (Paradis et al., 2004).

#### 2.4.2 Drought tolerance

A generalized linear mixed‐effects model was used to determine whether larval survival rates varied between sites in association with site characteristics. The model was fitted with a binomial error structure and with host plant drought treatment and percentage chalk cover (geology) as fixed effects with an interaction term, and population as a random intercept (Equation 2). The slope angle was not included due to a 0.8 Pearson's correlation with chalk cover.

(2) S=T+G+T.G+p+ε,

where *S* is the larval survival rate, *T* is the treatment (drought/control), *G* is the geology of the origin site (percentage cover chalk grassland), *p* is a random intercept for the origin population of the larvae, and *ε* indicates error term with zero mean and normal distribution.

A series of model simplifications were carried out (removal of the interaction term, removal of geology variable, and removal of treatment variable), and all versions of the model were compared using the *model.sel* function from the R package MuMIn (Barton, 2020).

#### 2.4.3 Population genetics

Measures of genetic diversity and differentiation (based on 287 individuals from 15 sites; Figure 2), including Wright's *F* statistics, heterozygosity, allelic richness, and effective population sizes were carried out using GenePop v4.7.0 (Rousset, 2008), FSTAT v2.9.4 (Goudet, 1994), Arlequin v3.5 (Excoffier & Lischer, 2010), NeEstimator v2 (Do et al., 2014), and PopGenReport (Adamack & Gruber, 2014).

Population structure was estimated using STRUCTURE v.2.3.4 (Falush et al., 2007; Pritchard et al., 2000), using an admixture model and correlated allele frequencies with a 100,000 burn‐in and 1,000,000 MCMC replications per chain. The potential number of genetic clusters (*K*) was tested from one to six, with 20 chains run per *K*. The likeliest *K* within the sample sets was estimated using the program STRUCTURE Harvester (Earl & VonHoldt, 2012) and visualized using CLUMPAK (Kopelman et al., 2015). Four separate STRUCTURE runs were conducted: (a) all individuals allocated by the population from which they were sampled (15 populations, *n* = 287), (b) all chalk site and all nonchalk sites grouped into two populations (*n* = 137 and 150, respectively), (c) only the individuals from the eight chalk sites (*n* = 137), and iv) only the individuals from the seven nonchalk sites (*n* = 150).

Individuals were then pooled by site to generate allele frequencies for genetic distance analysis. Weir and Cockerham pairwise *F*_ST_ values were calculated using Genepop. Mean allelic richness across all loci for each site was calculated using FSTAT. A Mann–Whitney *U* test was carried out to compare the allelic richness of individuals on chalk with nonchalk sites. Pairwise *F*_ST_ values were calculated for each site pair combination, with each combination assigned to one of three categories based upon the individual geologies of the two sites: (a) both chalk, (b) both nonchalk, (c) one chalk, and the other nonchalk. The slope angle was not included owing to all nonchalk sites being shallow and all but one of the chalk sites steep. Pairwise *F*_ST_ values were fitted into a linear regression with geology and Euclidean distance between sites as a fixed effect (Equation 3).

(3) F=G+D+ε,

where *F* is the pairwise *F*_ST_ score between each pair of sites, *G* is the site geology (chalk/nonchalk), *D* is the Euclidian distance between sites, and *ε* indicates error term with zero mean and normal distribution.

As pairwise *F*_ST_ values between sites are not independent, Mantel randomization tests with 999 permutations were conducted to assess whether the predictor variable (geology) was significant following the methodology described in Powney et al. (2012). The number of significantly different groupings within site type pairs was determined via a Tukey HSD test.

## 3 RESULTS

### 3.1 Phenology

**FIGURE 3:**
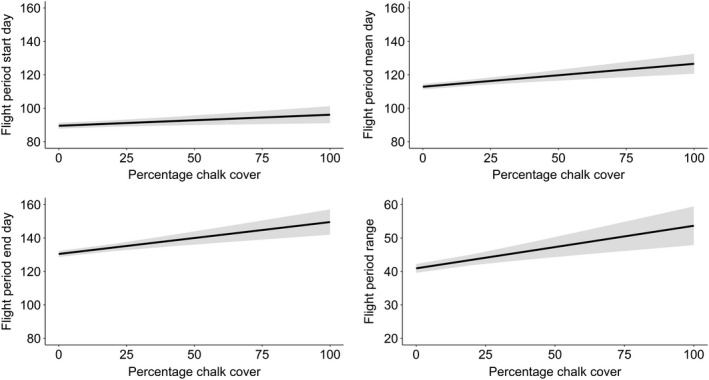
Estimated model values from Equation (1) for four measures of phenology for *M*. *jurtina* in relation to percentage cover of chalk

**FIGURE 4:**
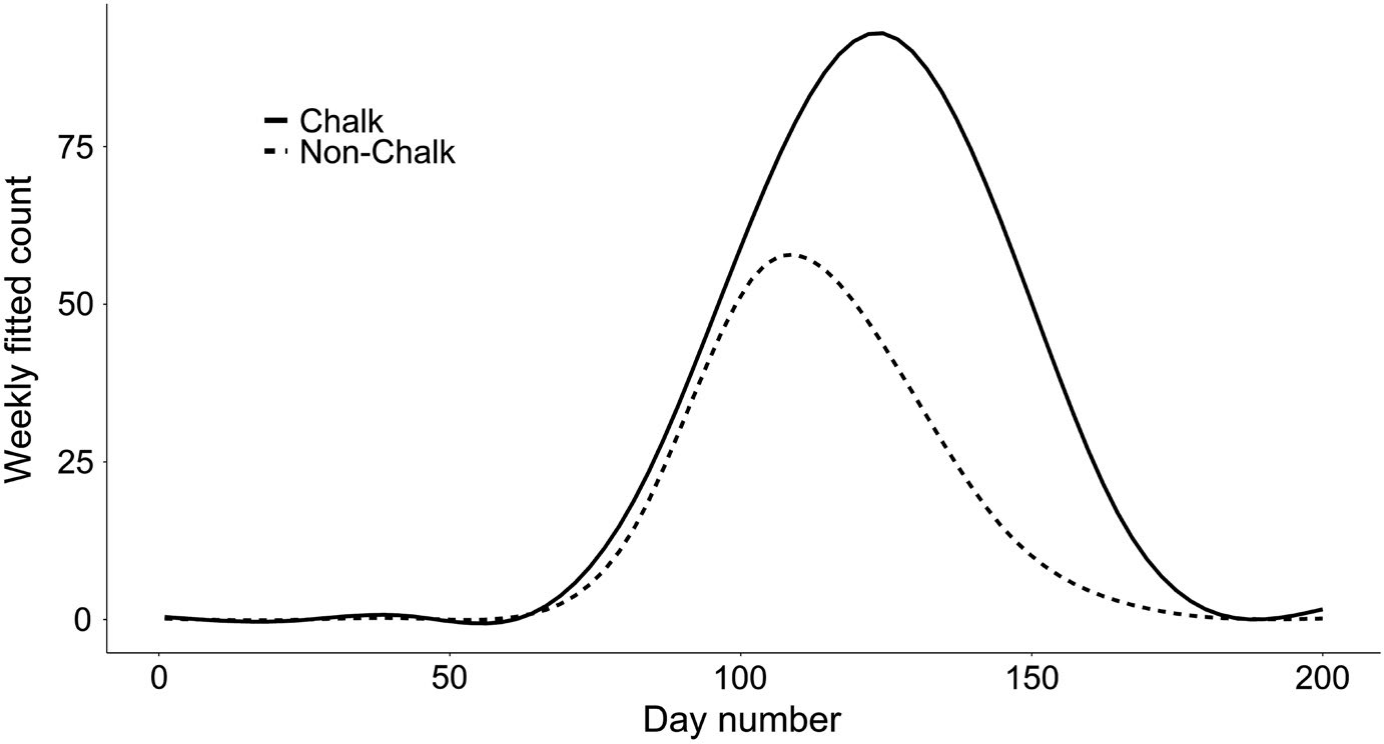
Annual mean weekly fitted counts for *M*. *jurtina* between 1976–2015, across UKBMS sites split by chalk presence, where chalk sites are classified as sites with a greater than 0% cover of chalk within 500 m of the site centroid. Day number 1 = 1st April i.e. the start of the UKBMS recording window

**FIGURE 5:**
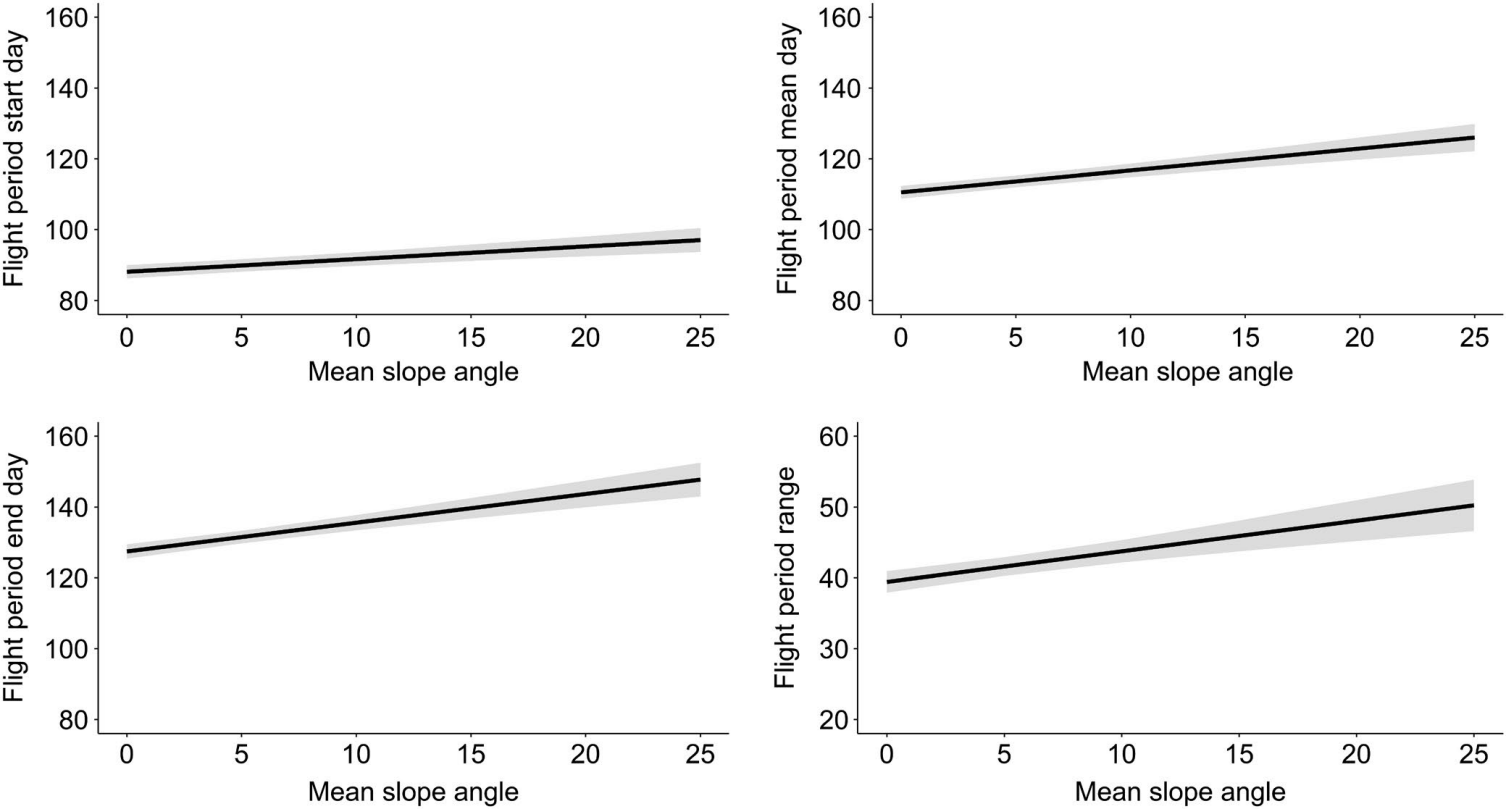
Estimated model values from Equation (1) for four measures of phenology for *M*. *jurtina* in relation to site mean slope angle

**FIGURE A1:**
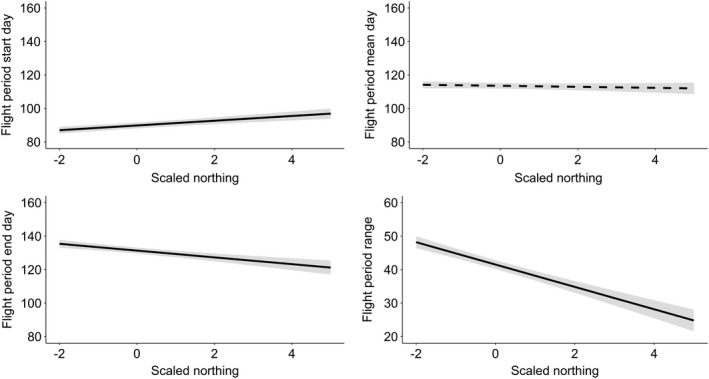
Estimated model values from Equation (1) for four measures of phenology for *M*. *jurtina* in relation to Scaled Northing. Scaled Northing had a significant effect (solid lines) on all measures of phenology, with the exception of mean dates (dashed line)

**FIGURE A2:**
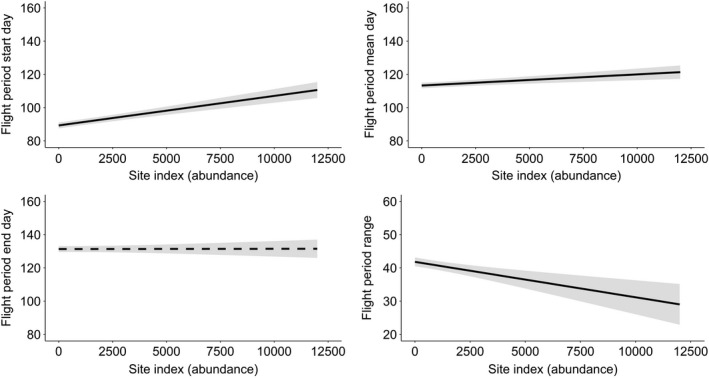
Estimated model values from Equation (1) for four measures of phenology for *M*. *jurtina* in relation to mean butterfly abundance at each site. Abundance had a significant effect (solid lines) on all measures of phenology with the exception of flight period end dates (dashed line)

All phenology measures were significantly positively associated with differences in chalk cover (flight start date coefficient = 0.07, *p* = .009; mean date coefficient = 0.14, *p* < .001; end date coefficient = 0.19, *p* < .001; flight period range coefficient = 0.13, *p* < .001, Figures 3 and 4, Appendix 2, Table A4) and mean slope angle (start date coefficient = 0.36, *p* < .001; mean date coefficient = 0.62, *p* < .001; end date coefficient = 0.81, *p* < .001; flight period range coefficient 0.43, *p* < .001 Figure 5, Appendix 2, Table A4). Hence, average flight period dates were later on sites with greater levels of chalk cover or steeper slope angles, and average flight periods were longer on sites with greater levels of chalk cover or steeper slope angles. Northing and abundance were also significantly associated with all four measures of phenology, with two exceptions: (a) northing was not associated with flight period mean date and (b) mean local abundance was not associated with the flight period end date (Appendix 2, Table A4). Estimated model values for Equation (1) regarding abundance and northing can be found in Figures A1 and A2. Aspect, altitude, and easting were not significantly associated with any measure of phenology; however, aspect and altitude were both marginally significantly associated with the flight period range (flight period range coefficient = −0.52, *p* = .08 and flight period range coefficient = 0.01, *p* = .09, respectively). The residuals from each model showed no evidence of spatial autocorrelation using Moran's I test (start day model I: observed (*O*) = 0.001, expected (*E*) = −0.0001, *SD* = 0.001, *p* = .259; Mean day model I: *O* = 0.0007, *E* = −0.0002, *SD* = 0.001, *p* = .476; End day model I: *O* = 0.0008, *E* = −0.0002, *SD* = 0.001, *p* = .421; Range model I: *O* = 0.001, *E* = −0.0002, *SD* = 0.001, *p* = .277).

### 3.2 Drought tolerance

**FIGURE 6:**
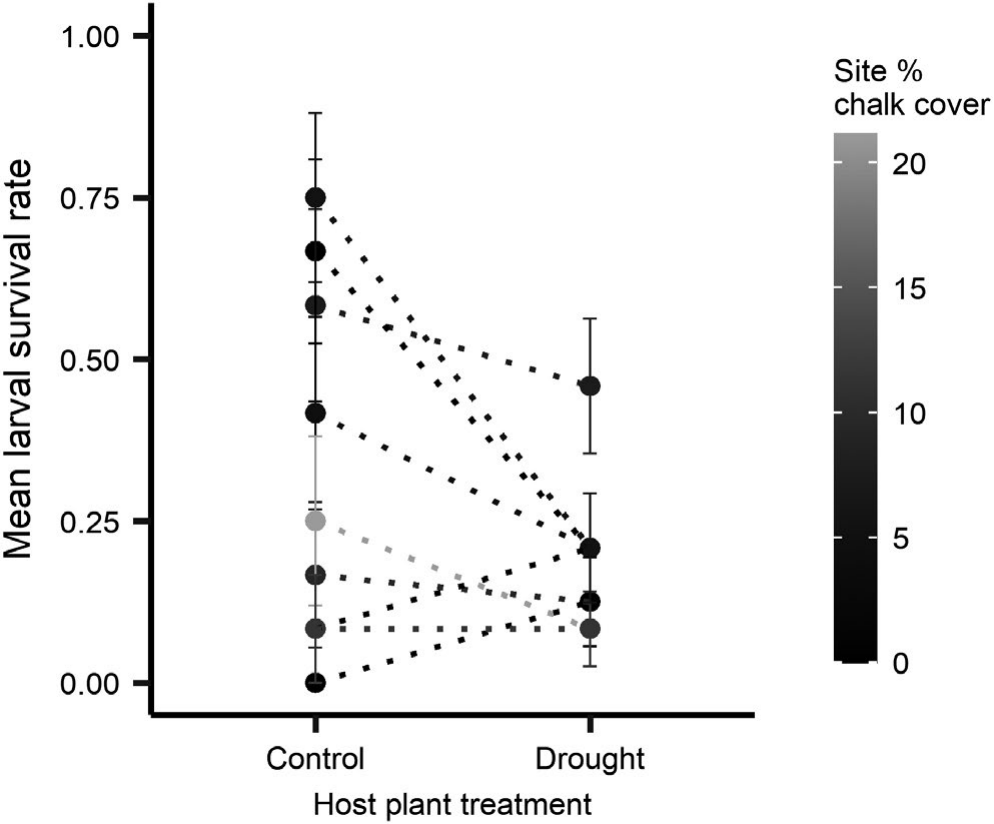
Mean survival rates of *M*. *jurtina* larvae when reared on control and drought‐stressed host plants. Populations are coloured by percentage chalk cover at each site, however chalk cover had no significant effect on larval survival rates

Model simplification determined that the best fitting model did not include chalk cover as a fixed effect [AICc 377.9 vs. 339.5 (treatment and geology additive), 341.4 (treatment and geology interaction), and 346.1 (geology only)], that is, larval survival rates were significantly affected by host plant drought treatment (intercept = 0.78, *SE* = 0.33, *z*‐value = 2.36, *p* = .018; drought coefficient = −0.84, *SE* = 0.28, *z*‐value = 2.97, *p* = .003; Figure 6), but chalk cover had no effect on larval survival rates.

### 3.3 Population genetics

**FIGURE 7:**
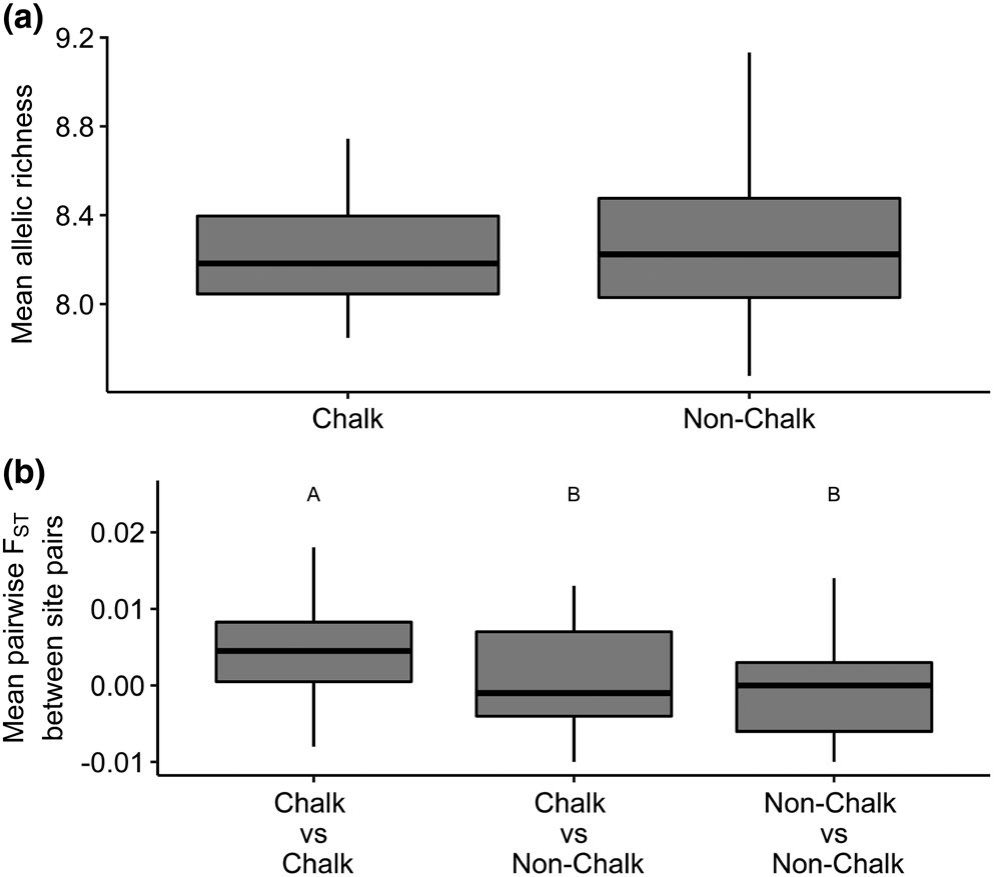
The effects of site geology versus two measures of genetic diversity: (a) allelic richness (b) pairwise *F*_ST_, letters indicate significance groupings

**FIGURE A3:**
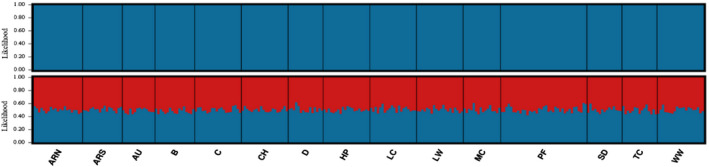
STRUCTURE individual assignment bar plots for *M*. *jurtina* individuals within the study area. Individuals split by site on the *x* axis and likelihood of assignment of the individual into genetic clusters on the *y*. Colours indicate different genetic clusters. Plot A: *K* = 1, Plot B: *K* = 2. All individuals have a roughly 50% chance of being assigned into either of the clusters where *K* = 2, indicating no apparent population structuring, i.e. no individuals are more or less likely to be assigned to any *K*, therefore *K* = 1

All populations within the 15 sites in southern England displayed high levels of genetic diversity and low levels of genetic differentiation (Appendix 3, Tables A5–A9, Figure A3). In summary, no linkage disequilibrium occurred between any pair of loci (Table A5). Null allele frequencies were <0.2 for all site loci combinations, except for Mj4870 at ARS (Table A6). The microsatellites used displayed a high level of variability (*H*_O_ = 0.279–0.902), and no locus displayed significant heterozygote excess or deficit (Table A7). No *F*_ST_ values per locus were significantly different from zero; however, F_IS_ values were significant at four of the six loci (Table A7). All populations displayed a high level of heterozygosity, with high levels of allelic richness and infinite estimated effective population sizes (Table A8). Allelic richness was not significantly affected by site geology (*p* = .867), with a mean allelic richness of 8.2 for chalk sites and 8.3 for nonchalk sites (Figure 7).

Pairwise *F*_ST_ scores between pairs of sites were extremely low (mean = 0.002, variance = 0.00004), and none were significantly greater than zero (Table A9). However, when site pairs were grouped by geology (i.e., chalk and chalk, nonchalk and nonchalk, and chalk and nonchalk), combinations within site pairs had a significant effect on pairwise *F*_ST_ (Table 1, Figure 7), indicating evidence of weak population differentiation. The distance between sites had no effect on pairwise *F*_ST_ (Table 1). No evidence of the population structure was found between these 15 populations. No population was found to be strongly genetically distinct from any other population, regardless of the number or allocation of sites included in the analysis (Figure A3).

**TABLE 1:** Effects of site pair geology and distance between sites on pairwise *F*_ST_ (Equation 3)

Model	Response	Factor	Degrees of freedom	Sum of squares	Mean square	*F* value	*p*‐value
Equation (3)	*F*_ST_	Geology	2	0.0003	0.0005	3.8105	.025
Equation (3)	*F*_ST_	Distance	1	0.000009	0.000009	0.243	.623
Equation (3)	*F*_ST_	Residuals	101	0.004	0.00004	NA	NA

## 4 DISCUSSION

In this study, we quantified characteristics of *M*. *jurtina* flight periods with respect to geology and topography. We also determined whether differences in other aspects of ecology (population genetics and drought tolerance) were also associated with the same landscape attributes. We found significant, positive, associations between the phenology of *M*. *jurtina* and geology (chalk grassland) and topography (steepness of sites being a general proxy for topographical heterogeneity), that is, key flight dates are delayed with increasing chalk cover and slope angle. These associations remained after accounting for abundance, therefore, aspects of geology and topography are associated with phenology independent of mean local abundance. We found no strong evidence of genetic structuring of *M*. *jurtina* populations linked to geology, and only very weak evidence of genetic differentiation among populations. Finally, we found no effect of geology on larval survival (drought response).

Microclimatic heterogeneity may explain the longer flight periods on steeper (more topographically diverse) chalk grasslands. Habitat and topographic diversity can allow species to persist in areas of suitable microclimate when the surrounding climate is no longer favorable (Bennie et al., 2008), and habitat heterogeneity has been promoted as a method of improving species resilience under climate change (Crick et al., 2020). For example, south‐facing chalk grassland hillsides were found to harbour populations of the warmth loving species the silver‐spotted skipper (*Hesperia comma*), absent from other habitat types (Davies et al., 2006). However, increasing ambient temperatures at sites due to climate change has seen an expansion in the local distributions of this species (Lawson et al., 2014; Wilson et al., 2010). If microclimate heterogeneity alone causes the longer flight periods, we might expect to see a two‐tailed expansion to the flight period on steep chalk sites, with suitable habitat patches available earlier as well as later in the year.

In contrast, we found that all measures of phenology were positively associated with chalk cover, including start date. This means that sites with more chalk have later start dates, creating a long, single‐tailed extension to the flight period later into the season. Similar results were found regarding topography. These results indicate that phenological differences are likely not a simple effect of either warmer conditions during the summer or the heterogeneous nature of chalk grassland sites and the range of microclimates available (Diacon‐Bolli et al., 2012).

Flight start dates are typically a result of the effects of spring temperatures on larval development (Roy & Sparks, 2000); therefore, drought conditions on steep chalk grassland sites may additionally impact larval development and hence adult phenology. Drought conditions have been shown to lead to lengthened larval development times and later emergence dates, in species such as the speckled wood, as a result of physiological stress (Gibbs et al., 2012). In habitats with heterogeneous microclimates, such as hilly chalk grasslands, certain microhabitats (e.g., with thinner soils on south‐facing slopes) may lead to host plants becoming particularly drought‐stressed. This would result in a certain proportion of individuals at a site with delayed emergences and a more protracted flight period overall but one that is single‐tailed. One point to note is that the fixed effects in our models account for relatively little variation within the data (7%–15%), and the majority of variation (46%–77%) is explained by the random effects for site and year. This is unsurprising as year captures weather effects, which are known to have a large effect on butterfly phenology (Mills et al., 2017; Roy et al., 2001), although there may be differences between sites that are not captured in our relatively coarse scale topographic descriptors (e.g., local vegetation and microclimatic factors that further mediate phenological responses; Davies et al., 2006; Hindle et al., 2015).

A limitation of this study is that UKBMS data do not fully encompass the flight period of *M*. *jurtina*. Protracted flight periods into October have been reported on these southern chalk grassland sites (Thomas & Lewington, 2010), whereas UKBMS recording runs from the start of April until the end of September. Therefore, it is feasible that flight period end dates on some sites are later compared with those used in this analysis, and we may underestimate the protracted phenology of *M*. *jurtina* on steeper sites with more chalk substrate. Additionally, differences in local vegetation characteristics and/or fine scale topographic variation could be having effects that are not accounted for in this analysis.

Our molecular analysis results support those of Richard et al. (2015) and Villemey et al. (2016) in finding high levels of genetic diversity within *M*. *jurtina* populations and low levels of genetic divergence between populations using microsatellite markers. These results are consistent with those of other studies, although not directly comparable due to the use of differing techniques (allozymes and AFLPs) (Baxter et al., 2017; Goulson, 1993b; Habel et al., 2009; Schmitt et al., 2005; Thomson, 1987). Despite being statistically significant, the differences in genetic differentiation between site types (as indicated by pairwise *F*_ST_ scores) are extremely low, being below the 0.05% threshold typically viewed as indicative of genetic differentiation (Freeland, 2011). This suggests that populations on chalk sites are marginally more genetically distinct from populations on other chalk sites than those from populations in the surrounding environment. Additionally, no population structuring was found via any combination of sites, possibly due to the dispersal ability of *M*. *jurtina* (Schneider et al., 2003) and the ubiquity of its host plants. Therefore, it appears that all populations included in the study belong to a single, large population, with properties similar to the one at panmixia with random mating. Very low levels of differentiation are present, although insufficient to have any great effect on population structuring. The suggestion that populations of *M*. *jurtina* on chalk grasslands form a distinct genetic race is not supported; in fact, the opposite is found, with populations on chalk sites being more distinct from each other, although these levels of differentiation are very low. Therefore, we conclude that the differential phenology associated with geology and topography found in this study is unlikely to be explained by differentially adapted host races. However, it should be noted that due to the high correlation found between chalk percentage cover and site steepness, we cannot determine the effect of topography with this experimental setup. Therefore, caution in interpretation is required as our other analyses have shown that site topography can have an effect on aspects of *M*. *jurtina* ecology.

Contrary to our expectations, we found no association between the percentage of chalk cover from source sites and larval survival when exposed to drought conditions. However, these results should be interpreted with caution owing to the relatively small sample size and spatial scale of the analysis, and the fact that slope could not be included in the drought models, despite affecting phenology. Additionally, in wild situations, larvae would be able to move from plant to plant, ensuring that a sufficient quantity of food could be consumed. In the experimental setup, larvae were constrained to single pots containing food plants and therefore unable to move to fresh sites, even though sufficient green plant material was available throughout the experiment and remained at the end to ensure that food quantity was not a limiting factor in larval growth. Our results suggest that although drought conditions reduce larval survival rates, the effects do not appear to be mitigated by local adaptation specific to chalk sites.

In conclusion, we found butterfly phenology varied at the national scale associated with geology and topography. We found neither evidence of genetic structuring of populations based upon these site conditions nor any differences in drought tolerance. Future research may benefit from a detailed analysis of other ecological factors influencing phenology such as host plant distribution and quality at different sites. This may allow a greater understanding of why phenology is affected by both chalk percentage cover and site topography. Additionally, factors affecting the potential for local adaptation could also be investigated, for example, slope aspect, microclimate, vegetation cover, and habitat management (Bennie et al., 2006; Brakefield, 1987; van Noordwijk et al., 2012). Such studies will become increasingly important for understanding and predicting species responses to a rapidly changing climate.

## CONFLICT OF INTEREST

We declare no conflicts of interest.

## AUTHOR CONTRIBUTIONS

**Matthew P. Greenwell:** Data curation (equal); formal analysis (lead); investigation (lead); methodology (equal); project administration (equal); writing‐original draft (lead); writing‐review & editing (equal). **Marc S. Botham:** Conceptualization (supporting); data curation (equal); writing‐review & editing (equal). **Michael W. Bruford:** Conceptualization (equal); formal analysis (supporting); investigation (supporting); methodology (supporting); supervision (equal); writing‐review & editing (equal). **John C. Day:** Conceptualization (equal); formal analysis (supporting); investigation (supporting); methodology (equal); supervision (supporting); writing‐review & editing (equal). **Luke C. Evans:** Formal analysis (supporting); methodology (supporting); writing‐review & editing (equal). **Melanie Gibbs:** data curation (equal); formal analysis (equal); investigation (equal); methodology (equal); writing‐review & editing (equal). **Ian Middlebrook:** Data curation (equal); writing‐review & editing (equal). **David B. Roy:** Conceptualization (supporting); data curation (equal); writing‐review & editing (equal). **Kevin Watts:** Conceptualization (equal); funding acquisition (supporting); investigation (supporting); methodology (equal); supervision (equal); writing‐review & editing (equal). **Tom H. Oliver:** Conceptualization (lead); formal analysis (supporting); funding acquisition (lead); investigation (supporting); methodology (supporting); project administration (supporting); supervision (lead); writing‐original draft (supporting); writing‐review & editing (equal).

## Supporting information

Supplementary Material

Supplementary Material

## Data Availability

Drought tolerance data are available in the Environmental Information Data Centre (EIDC) at the NERC Centre for Ecology & Hydrology. DOI: https://doi.org/10.5285/f26f391f‐a17b‐4a0d‐85c7‐ab8af85c3f1b. Genotype data are available in Mendeley Data. https://doi.org/10.17632/kfz2fbrkdx.1. The UKBMS Site Index data and Natural England Priority Habitat data are publicly available and referenced. The weekly fitted values for *M*. *jurtina* are supplied in the Supplementary Materials, along with the site number allocations used. This is a subset of a full dataset for all UK butterfly species which is available upon request from the UKBMS. All data used in this study have been made available.

## APPENDIX 1 Site characteristics and correlations

### 1.1

**TABLE A1:** Pearson's Rank Correlation coefficients for site characteristics calculated using all 539 UKBMS sites used in the analyses

Variable 1	Variable 2	Correlation	*p* value	*t* value
Chalk Area	Slope Mean	0.108	<.001	4.032
Chalk Area	Slope *SD*	0.095	<.001	3.554
Chalk Area	Mean Aspect (North)	0.011	.683	0.409
Chalk Area	Aspect (North) *SD*	0.015	.577	0.557
Chalk Area	Mean Aspect (East)	−0.056	.038	−2.08
Chalk Area	Aspect (East) *SD*	−0.017	.522	−0.641
Chalk Area	Mean Altitude	−0.063	.019	−2.353
Chalk Area	Altitude *SD*	−0.032	.234	−1.19
Chalk Area	Northing	−0.048	.073	−1.796
Chalk Area	Easting	0.081	.003	3.029
Mean Slope Angle	Slope *SD*	0.763	<.001	43.9
Mean Slope Angle	Mean Aspect (North)	−0.017	.522	−0.64
Mean Slope Angle	Aspect (North) *SD*	0.007	.06	0.271
Mean Slope Angle	Mean Aspect (East)	0.005	.843	0.199
Mean Slope Angle	Aspect (East) *SD*	0.017	.539	0.614
Mean Slope Angle	Mean Altitude	0.483	<.001	20.52
Mean Slope Angle	Altitude *SD*	0.582	<.001	26.62
Mean Slope Angle	Northing	0.064	.018	2.369
Mean Slope Angle	Easting	−0.387	<.001	15.64

Note

Each correlation calculated with 1,381 degrees of freedom.

**TABLE A2:** Pearson's Rank Correlation coefficients for site characteristics calculated using the 15 sites used in the genetic and drought analyses

Variable 1	Variable 2	Correlation	*p* value	*t* value
Chalk Area	Slope Mean	0.811	<.001	5.006
Chalk Area	Slope *SD*	0.667	.007	3.237
Chalk Area	Mean Aspect (North)	0.452	.091	1.829
Chalk Area	Aspect (North) *SD*	−0.265	.339	−0.992
Chalk Area	Mean Aspect (East)	−0.051	.857	−0.184
Chalk Area	Aspect (East) *SD*	0.094	.739	0.34
Chalk Area	Mean Altitude	0.357	.192	1.377
Chalk Area	Altitude *SD*	0.528	.043	2.242
Chalk Area	Northing	0.225	.421	0.832
Chalk Area	Easting	0.152	.588	0.556
Mean Slope Angle	Slope *SD*	0.889	<.001	7.012
Mean Slope Angle	Mean Aspect (North)	0.335	.223	1.281
Mean Slope Angle	Aspect (North) *SD*	0.067	.813	0.241
Mean Slope Angle	Mean Aspect (East)	−0.209	.455	−0.77
Mean Slope Angle	Aspect (East) *SD*	−0.155	.582	−0.565
Mean Slope Angle	Mean Altitude	0.613	.015	2.798
Mean Slope Angle	Altitude *SD*	0.587	.022	2.611
Mean Slope Angle	Northing	0.447	.095	1.803
Mean Slope Angle	Easting	0.455	.089	1.841

Note

Correlations calculated with 13 degrees of freedom.

**TABLE A3:** Landscape attribute data for each of the fifteen sites used in the genetic and drought analyses

Site	% Chalk grass cover	Slope angle		Aspect (East)		Aspect (North)		Altitude		Northing	Easting
		Mean	*SD*	Mean	*SD*	Mean	*SD*	Mean	*SD*		
ARN	9.39	10.77	5.19	−0.26	0.64	0.32	0.65	129.16	49.66	197,086	472,827
ARS	14.16	9.29	5.04	−0.48	0.51	0.44	0.56	130.88	49.34	196,060	472,285
AU	14.56	5.39	3.32	0.38	0.77	0.24	0.46	101.4	41.96	183,700	454,500
B	0	3.04	3.03	0.28	0.49	−0.03	0.83	108.23	30.73	165,000	450,800
C	0	1.59	0.89	0.05	0.74	0.46	0.5	110.88	40.92	151,900	466,300
CH	7.56	8.18	6.42	−0.56	0.59	0.27	0.52	134.8	50.09	206,700	484,700
D	4.7	8.9	3.87	0.35	0.36	0.18	0.85	141.69	45.81	209,500	490,000
HP	0	0.69	0.47	−0.54	0.75	−0.03	0.39	90.82	45.9	190,000	461,500
LC	21.17	10.89	3.92	0.31	0.62	0.61	0.39	96.71	39.17	180,900	458,700
LW	0	3.71	1.82	−0.27	0.49	−0.28	0.79	68.79	22.32	192,300	456,200
MC	0	0.45	0.62	0.37	0.75	0.32	0.46	83.89	34.73	174,100	463,700
PF	0	1.27	0.9	0.5	0.33	−0.24	0.77	87.31	29.87	161,000	461,500
SD	11.46	7.1	3.53	−0.23	0.42	0.09	0.88	117.68	52.23	191,500	467,500
TC	5.73	4.74	3.21	0.59	0.37	0.34	0.63	144.72	44.29	208,800	490,400
WW	9.39	1.55	1.42	0.01	0.5	0.61	0.61	80.17	19.97	209,631	446,434

Note

Data compiled from Natural England priority habitat maps and a 50 m resolution digital elevation map (Morris & Flavin, 1990). Slope Angle = Degrees from horizontal, such that 0 = flat, 90 = vertical. Aspect (East) = Mean Eastness of aspect in landscape around site (Eastness = sin((aspect × PI)/180), such that 1 = due East, −1 = due West). Aspect (North) = Mean Northness of aspect in landscape around site (Northness = cos((aspect × PI)/180), such that 1 = due North, −1 = due South). Altitude = Mean height above sea level (m).

## APPENDIX 2 Phenology

### 2.1

**TABLE A4:** Linear mixed effects model outputs for Equation (1), showing the effects of geology, topography, site abundance and Scaled Northing on measures of *M*. *jurtina* phenology

Response variable	Marginal R^2^	Conditional R^2^	Fixed effects	Estimate	Lower CI	Upper CI	Standard error	Degrees of freedom	*t* value	*p*‐value
Start Date	0.07	0.61	Intercept	87.1	85.12	89.11	1.01	70.32	85.99	<.001
			Chalk cover %	0.07	0.02	0.12	0.03	414.36	2.61	.009
			Slope Angle	0.36	0.22	0.5	0.07	420.69	5	<.001
			Abundance	0.002	0.001	0.002	0.0002	4,563.85	8.97	<.001
			Northing	1.42	0.92	1.92	0.26	444.28	5.53	<.001
			Easting	0.1	−0.46	0.66	0.29	449.52	0.36	.722
			Aspect	0.05	−0.44	0.53	0.25	447.78	0.18	.854
			Altitude	0.002	−0.008	0.01	0.005	470.35	0.38	.703
Mean Date	0.12	0.77	Intercept	109.07	107.1	111.05	1.01	95.37	108.17	<.001
			Chalk cover %	0.14	0.08	0.2	0.03	460.92	4.51	<.001
			Slope Angle	0.62	0.45	0.79	0.09	467.64	7.2	<.001
			Abundance	0.001	0.0004	0.001	0.0002	5,624.12	4.1	<.001
			Northing	−0.31	−0.9	0.29	0.3	479.18	−1.01	.315
			Easting	0.35	−0.32	1.01	0.34	486.01	1.02	.31
			Aspect	−0.29	−0.87	0.29	0.3	485.15	−0.96	.336
			Altitude	0	−0.003	0.019	0.01	500.45	1.38	.169
End Date	0.15	0.71	Intercept	125.67	123.43	127.91	1.14	132.1	109.82	<.001
			Chalk cover %	0.19	0.12	0.27	0.04	451.12	4.89	<.001
			Slope Angle	0.81	0.6	1.03	0.11	458.21	7.38	<.001
			Abundance	0.00001	−0.0004	0.0005	0.0002	5,534.54	0.06	.952
			Northing	−2.03	−2.79	−1.27	0.39	472.11	−5.19	<.001
			Easting	0.29	−0.56	1.15	0.44	479.23	0.67	.503
			Aspect	−0.5	−1.25	0.24	0.38	478.09	−1.32	.188
			Altitude	0.01	−0.003	0.03	0.008	495.19	1.53	.127
Range	0.13	0.47	Intercept	38.37	36.67	40.06	0.87	146.56	44.24	<.001
			Chalk cover %	0.13	0.07	0.19	0.03	379.49	4.27	<.001
			Slope Angle	0.43	0.27	0.6	0.09	383.69	5.11	<.001
			Abundance	−0.001	−0.002	−0.001	0.0003	3,703.51	−4.03	<.001
			Northing	−3.34	−3.93	−2.75	0.3	412.6	−11.002	<.001
			Easting	0.16	−0.51	0.83	0.34	414.29	0.47	.64
			Aspect	−0.52	−1.1	0.06	0.3	413.08	−1.76	.08
			Altitude	0.01	−0.002	0.02	0.006	437.7	1.68	.092

Note

Models are repeated for each measure of phenology.

## APPENDIX 3 Population genetics

### 3.1

**TABLE A5:** Composite linkage disequilibrium test outputs for all locus pair combinations, calculated in Genepop v4.7 (Rousset, 2008)

Locus pair		Chi2	*df*	*p*‐value
Mj7232	Mj5522	30.528	30	.439
Mj7232	Mj0247	23.029	26	.631
Mj5522	Mj0247	7.734	26	1
Mj7232	Mj4870	30.842	30	.423
Mj5522	Mj4870	15.522	30	.986
Mj0247	Mj4870	20.015	26	.791
Mj7232	Mj7132	16.554	30	.978
Mj5522	Mj7132	23.531	30	.793
Mj0247	Mj7132	13.529	26	.979
Mj4870	Mj7132	23.315	30	.802
Mj7232	Mj5331	24.854	26	.527
Mj5522	Mj5331	14.212	26	.97
Mj0247	Mj5331	13.037	22	.932
Mj4870	Mj5331	17.472	26	.894
Mj7132	Mj5331	11.868	26	.992

**TABLE A6:** Locus by population estimated null allele frequencies for all sites per locus

Locus	ARN	ARS	AU	B	C	CH	D	HP	LC	LW	MC	PF	SD	TC	WW	Mean
Mj7232	0.028	0.145	0	0.137	0.079	0.096	0.022	0.032	0.086	0	0.171	0.05	0.048	0.013	0.032	0.063
Mj5522	0	0.055	0.048	0	0	0	0.03	0.055	0.006	0.013	0.029	0.021	0.076	0.074	0	0.027
Mj0247	0.042	0.03	0	0.085	0.018	0	0.041	0	0	0.037	0.111	0.064	0.068	0	0.048	0.036
Mj4870	0.075	0.205	0	0	0	0.064	0.086	0.003	0.159	0.151	0	0.113	0.15	0.189	0.128	0.088
Mj7132	0.022	0.065	0	0.045	0	0	0.091	0	0	0.028	0	0	0.047	0.002	0	0.02
Mj5331	0	0	0	0.032	0	0	0	0	0.03	0.028	0.028	0.027	0	0	0	0.01

Note

Values in bold exceed 0.2 frequency of null alleles. Null allele frequencies calculated in Genepop v4.7 (Rousset, 2008).

**TABLE A7:** Population‐wide expected and observed heterozygosity, and percent difference ((*E* − *O*)/*E ** 100), *F*_IT_, *F*_IS_ *F*_ST_ and at each locus. Bartlett's *K*‐squared: 0.03, *df* = 1, *p*‐value = .8618. He, HO and percentage differences calculated in PopGenReport (Adamack & Gruber, 2014)

Locus	Number of samples	Number of alleles	He	Ho	He versus Ho % difference	FIT (*p*‐value)		FST (*p*‐value)		FIS (*p*‐value)	
Mj7232	285	12	0.798	0.762	−4.488	0.048	0.035	0.002	0.999	0.046	0.049
Mj5522	281	12	0.862	0.809	−6.226	0.064	0.008	0	1	0.064	0.006
Mj0247	283	31	0.941	0.842	−10.614	0.105	0	0	1	0.105	0
Mj4870	282	6	0.37	0.279	−24.596	0.252	0	0	0.982	0.252	0
Mj7132	282	10	0.741	0.752	1.5	−0.013	0.692	0.007	0.911	−0.02	0.77
Mj5331	286	22	0.894	0.902	0.913	−0.007	0.667	0.002	1	−0.009	0.692
Mean	283	16	0.768	0.724	−7.252	0.075	–	0.002	–	0.073	–

Note

*F*_IT_, *F*_ST_ and *F*_IS_ values calculated in Arlequin v 3.5.2.2 (Excoffier & Lischer, 2010).

**TABLE A8:** Sample sizes, genetic diversity, allelic richness, number of private alleles and effective population sizes for *M*. *jurtina* populations in the south of England

Site	Sample size	Mean Hexp (*SD*)		Ar	Ap	Ne(1)	Ne(2)
All Sites	287	0.764	−0.215	–	9	–	–
Aston Rowant North (ARN)	21	0.76	−0.176	8.278	0	∞	∞
Aston Rowant South (ARS)	17	0.753	−0.224	7.765	0	∞	∞
Aston Upthorpe (AU)	14	0.713	−0.269	8.5	0	∞	∞
Bowdown Forest (B)	17	0.738	−0.246	8.039	0	∞	∞
Crabtree Plantation (C)	20	0.783	−0.199	8.444	0	∞	∞
Coombe Hill (CH)	20	0.805	−0.145	8.69	0	∞	∞
Dancersend (D)	15	0.776	−0.223	8.806	0	∞	∞
Howbery Park (HP)	20	0.786	−0.163	8.438	0	∞	∞
Lardon Chase (LC)	20	0.796	−0.176	9.096	2	∞	∞
Little Whittenham (LW)	20	0.784	−0.229	8.682	0	∞	∞
Moore Copse (MC)	16	0.736	−0.266	8.858	1	∞	∞
Pamber Forest (PF)	37	0.771	−0.223	9.03	5	∞	∞
Swyncombe Down (SD)	15	0.747	−0.279	8.773	0	∞	∞
The Crong (TC)	15	0.771	−0.213	8.286	1	∞	∞
Wytham Woods (WW)	20	0.745	−0.192	8.097	0	∞	∞

Note

All values are estimated on a per population basis. *H*_exp_ expected heterozygosity calculated in Arlequin, A_r_ = allelic richness calculated in FSTAT v2.9.4 (Goudet, 1994), Ap = Private alleles calculated in PopGenRport, Ne(1) = effective population size estimated using the heterozygote excess method calculated in NeEstimator V2 (Do et al., 2014), Ne(2) = effective population size estimated using linkage disequilibrium method, calculated in NeEstimator.

**TABLE A9:** Pairwise *F*_ST_ values between fifteen pairs of sites across the south of England in 2017

	ARN	ARS	AU	B	C	CH	D	HP	LC	LW	MC	PF	SD	TC	WW
ARN	–	0.037	0.229	0.333	0.036	0.515	0.459	0.207	0.26	0.014	0.034	0.035	0.073	0.02	0.003
ARS	0.004	–	0.29	0.073	0.06	0.142	0.45	0.387	0.14	0.103	0.336	0.062	0.414	0.053	0.019
AU	0.004	−0.003	–	0.897	0.39	0.388	0.412	0.714	0.211	0.289	0.68	0.522	0.866	0.326	0.33
B	0.013	0.006	−0.003	–	0.098	0.092	0.306	0.896	0.389	0.167	0.487	0.882	0.958	0.451	0.055
C	0.007	−0.003	0.003	0.005	–	0.37	0.275	0.73	0.545	0.766	0.263	0.09	0.788	0.146	0.018
CH	0.001	0.012	0.011	0.02	0.002	–	0.127	0.339	0.719	0.39	0.411	0.123	0.485	0.141	0.027
D	0.001	−0.011	0.002	0.001	−0.003	0.012	–	0.411	0.864	0.843	0.929	0.268	0.619	0.6	0.034
HP	0.003	−0.004	−0.003	−0.009	−0.006	0.003	−0.002	–	0.436	0.6	0.845	0.891	0.947	0.467	0.171
LC	0.006	0.003	0.013	0.004	−0.003	0.003	−0.01	−0.003	–	0.929	0.255	0.255	0.731	0.177	0.105
LW	0.012	0	0.008	0.012	−0.007	0.004	−0.008	0.002	−0.008	–	0.359	0.435	0.932	0.031	0.008
MC	0.009	−0.003	−0.005	−0.003	0.001	0.014	−0.012	−0.006	0.005	0.006	–	0.552	0.761	0.38	0.135
PF	0.009	0	−0.005	−0.004	0	0.011	−0.006	−0.006	0.002	−0.001	−0.006	–	0.917	0.565	0.01
SD	0.014	−0.004	−0.005	−0.007	−0.008	0.008	−0.002	−0.005	0.001	−0.009	−0.003	−0.005	–	0.428	0.149
TC	0.015	0.004	0.003	0.005	−0.004	0.003	−0.002	−0.003	0	0.005	0.006	−0.002	−0.002	–	0.451
WW	0.007	0.009	−0.005	0.012	0.011	0.011	0.01	0	0.008	0.015	0.003	0.008	0.007	0.005	–

Note

Values below the diagonal = *F*_ST_ scores. Values above the diagonal indicate significance level *p* values. *p*‐values obtained after 2,100 permutations, indicative adjusted nominal level (5%) for multiple comparisons is 0.000476. All values are non‐significant.
